# CRIA: An Interactive Gene Selection Algorithm for Cancers Prediction Based on Copy Number Variations

**DOI:** 10.3389/fpls.2022.839044

**Published:** 2022-03-21

**Authors:** Qiang Wu, Dongxi Li

**Affiliations:** College of Data Science, Taiyuan University of Technology, Taiyuan, China

**Keywords:** gene selection, correlation-redundancy analysis, interaction analysis, copula entropy, copy number variations (CNVs), cancers prediction

## Abstract

Genomic copy number variations (CNVs) are among the most important structural variations of genes found to be related to the risk of individual cancer and therefore they can be utilized to provide a clue to the research on the formation and progression of cancer. In this paper, an improved computational gene selection algorithm called CRIA (correlation-redundancy and interaction analysis based on gene selection algorithm) is introduced to screen genes that are closely related to cancer from the whole genome based on the value of gene CNVs. The CRIA algorithm mainly consists of two parts. Firstly, the main effect feature is selected out from the original feature set that has the largest correlation with the class label. Secondly, after the analysis involving correlation, redundancy and interaction for each feature in the candidate feature set, we choose the feature that maximizes the value of the custom selection criterion and add it into the selected feature set and then remove it from the candidate feature set in each selection round. Based on the real datasets, CRIA selects the top 200 genes to predict the type of cancer. The experiments' results of our research show that, compared with the state-of-the-art related methods, the CRIA algorithm can extract the key features of CNVs and a better classification performance can be achieved based on them. In addition, the interpretable genes highly related to cancer can be known, which may provide new clues at the genetic level for the treatment of the cancer.

## Introduction

The occurrence of many diseases is associated with genome structural variations. Human genome variations include single nucleotide polymorphisms (SNPs), copy number variations (CNVs), etc. The copy number variations refer to the amplification, deletion, and more complex mutations in the genome of DNA fragments longer than 1 kb in length (Redon et al., 2006). SNPs account for 0.5% of the human genome, and nearly 12% of the human genome often undergoes copy number variations (Redon et al., 2006). Copy number variations have become an important genomic variation, and their role in the pathogenesis of complex human diseases is still being revealed.

The close relationship between CNVs and diseases has been widely recognized. Numerous studies have demonstrated that not a few human diseases involved copy number variations that could change the diploid status of particular locus of the genome (Zhang et al., 2016). The Flierl research team found that the higher vulnerability of Parkinson's disease and stress sensitivity of neuronal precursor cells carry an α-synuclein gene triplication (Flierl et al., 2014). Grangeon et al. (2021) discovered that early-onset cerebral amyloid angiopathy and Alzheimer Disease (AD) were related to an amyloid precursor protein (App) gene triple amplification. Breunis et al. (2008) reported that the copy number variations of FCGR2C gene promoted idiopathic thrombocytopenic purpura. Zheng et al. (2017) found that the low copy number of FCGR3B was associated with lupus nephritis in a Chinese population. And Pandey et al. (2015) revealed that there was both direct and indirect evidence suggesting abnormalities of glycogen synthase kinase (GSK)-3β and β-catenin in the pathophysiology of bipolar illness and possibly schizophrenia (SZ). Moreover, several neuro-developmental relevant genes, such as A2BP1, IMMP2, and AUTS2, were reported with mutational CNVs (Elia et al., 2010). In 2006, a research team composed of researchers from the United Kingdom, Japan, the United States, Canada and other countries studied 270 individuals in 4 groups of the HapMap project, and constructed the first-generation copy number variations map of the human genome, and obtained 144 CNVs region (about 12% of the size of the human genome). Among them, 285 CNVs regions were related to the occurrence of known diseases (Redon et al., 2006). Compared with SNPs, CNVs regions contained more DNA sequences, disease sites and functional elements, which could provide more clues for disease research. The publication of this map has become an important tool for studying the complex structural variations of the human genome and human diseases.

Cancer is a kind of diseases which involves uncontrolled abnormal cell growth and can spread to other tissues (Du and Elemento, 2015). The formation and development of cancer are also associated with copy number variations (Frank et al., 2007). Van Bockstal et al. (2020) discovered that HER2 gene amplification had a relationship with a bad result in invasive breast cancer and the amplification of heterogeneous HER2 had been described in 5–41% of breast cancer. The experimental results of Buchynska et al. (2019) shown that assessment of copy number variations of HER-2/neu, c-MYC and CCNE1 genes revealed their amplification in the tumors of 18.8, 25.0 and 14.3% of endometrial cancer patients, respectively. Heo et al. (2020) pointed out that CNVs were related to the mechanism of lung cancer development through a comparative experiment. Moreover, Tian et al. (2020) found that CNVs of CYLD, USP9X and USP11 were significantly associated with the risk of colorectal cancer. A latest global cancer burden data released by the International Agency for Research on Cancer(IARC) of the WHO showed that the number of patients with new cancer and cancer deaths in China ranked first around the world with 4.57 million patients with new cancer and 3 million cancer deaths, accounting for 23.7 and 30%, respectively. It is of great significance to investigate cancer causes and its treatment. Because the gene expression patterns in cancer tumor have high specificity (Liang et al., 2020), studying the relationship between these genetic information and cancer can provide a new idea for investigating the causes of cancer and help in early cancer diagnosis.

However, few studies have utilized machine learning (ML) or deep learning (DL) methods to use copy number variations data for the prediction of various cancer types. Zhang et al. (2016) used the mRMR and IFS methods to select 19 features from the 24,174 gene features of the copy number variations data set, which contained a total of 3,480 samples of 6 cancer types. They applied the Dagging algorithm with ten-fold cross-validation to classify cancer. But the accuracy of final result only reached 75%. Liang et al. (2020) used CNA_origin for cancer classification on the same data set. CNA_origin was an intelligent combined deep learning network, which was composed of two parts—a stacked autoencoder and a one-dimensional convolutional neural network with multiscale convolutional kernels. CNA_origin eventually had an overall accuracy of 83.81% on ten-fold cross-validation. But it could not identify which gene features were more important and more closely associated with cancer classification.

Here, we present an improved novel computational algorithm named CRIA, which can successfully classify cancer based on the information of gene CNVs levels from the same dataset. CRIA can not only effectively perform dimensionality reduction operation on high-dimensional gene CNVs data, which can improve the efficiency of the experiment, but also selects specific gene features closely related to cancer, making it clear which genes are more important in cancer classification. And the final results had higher classification accuracy than the state-of-the-art methods.

The rest sections of this paper are structured as follows: Section Background describes the theoretical background and related work. Section The Proposed Method-CRIA introduces the collection of CNVs dataset, the implementation details and performance of the proposed algorithm. Section Results and Discussions demonstrates the experimental results on CNVs dataset and the performance comparison with the recent methods. In section Conclusions, we summarize the conclusions and point out our future work.

## Background

In section Information Theory, we introduce some basic information theory knowledge, which is the core of our proposed algorithm. Before proposing our algorithm, we summarize some related work on gene selection methods and point out their drawbacks in section Related Work.

### Information Theory

As early as 1948, Shannon's information theory had been proposed (Shannon, 2001), providing an effective method for measuring random variables' information. The entropy can be understood as a measure of the uncertainty of a random variable (Cover and Thomas, 1991). The greater the entropy of a random variable, the greater its uncertainty. If *X* = {*x*_1_, *x*_2_, …, *x*_*l*_} is a discrete random variable, its probability distribution is *p*(*x*) = *P*(*X* = *x*), *x* ∈ *X*. The entropy of *X* is defined as:

(1) $$\begin{array}{l} H\left(X\right)=-\overset{l}{\underset{i=1}{\sum }}p\left(x_{i}\right)\log p\left(x_{i}\right) \end{array}$$

where *p*(*x*_*i*_) is the probability of *x*_*i*_. Here the base of log is 2 and specified that 0 log 0 = 0.

If *Y* = {*y*_1_, *y*_2_, …, *y*_*m*_} is a discrete random variable, *p*(*x*_*i*_, *y*_*j*_) is the joint probability of *X* and *Y*. Then, their joint entropy is defined as:

(2) $$\begin{array}{l} H\left(X,Y\right)=-\overset{l}{\underset{i=1}{\sum }}\overset{m}{\underset{j=1}{\sum }}p\left(x_{i},y_{j}\right)\log p\left(x_{i},y_{j}\right) \end{array}$$

If the random variable *X* is in a given situation, the uncertainty measure of the variable *Y* can be defined by conditional entropy as follows:

(3) $$\begin{array}{l} H\left(Y|X\right)=H\left(X,Y\right)-H\left(X\right)=-\overset{l}{\underset{i=1}{\sum }}\overset{m}{\underset{j=1}{\sum }}p\left(x_{i},y_{j}\right)\log p\left(y_{j}|x_{i}\right) \end{array}$$

where *p*(*y*_*j*_|*x*_*i*_) is the conditional probability of *Y* under the condition of *X*.

**Definition 1:** Mutual information (MI) (Cover and Thomas, 1991) is a measure of useful information in information theory. It can be regarded as the amount of information shared by two random variables. MI can be defined as:

(4) $$\begin{array}{l} I\left(X;Y\right)=\overset{l}{\underset{i=1}{\sum }}\overset{m}{\underset{j=1}{\sum }}p\left(x_{i},y_{j}\right)\log\frac{p\left(x_{i},y_{j}\right)}{p\left(x_{i}\right)p\left(y_{j}\right)}\\ \;=H\left(X\right)+H\left(Y\right)-H\left(X,Y\right)=H\left(X\right)-H\left(X|Y\right) \end{array}$$

**Definition 2:** Conditional mutual information (CMI) (Cover and Thomas, 1991) can be defined as the amount of information that shared by variables *X* and *Y*, if a discrete random variable *Z* = {*z*_1_, *z*_2_, …, *z*_*n*_} is known.

(5) $$\begin{array}{l} \begin{array}{c} I\left(X;Y|Z\right)=\overset{l}{\underset{i=1}{\sum }}\overset{m}{\underset{j=1}{\sum }}\overset{n}{\underset{k=1}{\sum }}p\left(z_{k}\right)p\left(x_{i},y_{j}|z_{k}\right)\log\frac{p\left(x_{i},y_{j}|z_{k}\right)}{p\left(x_{i}|z_{k}\right)p\left(y_{j}|z_{k}\right)}\\ =H\left(Y|Z\right)-H\left(Y|X,Z\right) \end{array} \end{array}$$

**Definition 3:** Joint mutual information (JMI) (Cover and Thomas, 1991) measures the amount of information shared by a joint random variable (*X*_1_, *X*_2_, ⋯*X*_*q*_) and Y and it can be defined as:

(6) $$\begin{array}{l} \begin{array}{c} I\left(X_{1},X_{2},...,X_{q};Y\right)=\underset{x_{1}\in X_{1}}{\sum }\underset{x_{2}\in X_{2}}{\sum }\cdots \underset{x_{q}\in X_{q}}{\sum }\underset{y\in Y}{\sum }p\left(x_{1},x_{2},...,x_{q},y\right)\\ \log\frac{p\left(x_{1},x_{2},...,x_{q},y\right)}{p\left(x_{1},x_{2},...,x_{q}\right)p\left(y\right)}\\ =H\left(X_{1},X_{2},...,X_{q}\right)-H\left(X_{1},X_{2},...,X_{q}|Y\right) \end{array} \end{array}$$

**Definition 4:** Interaction gain (IG) had been introduced by Jakulin (2003), Jakulin and Bratko (2004) to measure the amount of information shared by three random variables at the same time. Mutual information can be regarded as a two-way interaction gain. IG is defined as follows:

(7) $$\begin{array}{l} IG\left(X;Y;Z\right)=I\left(X;Y;Z\right)=I\left(X,Y;Z\right)-I\left(X;Z\right)-I\left(Y;Z\right) \end{array}$$

### Related Work

The irrelevant features and redundant features existed in high-dimensional data will damage the performance of the learning algorithm and reduce the efficiency of the learning algorithm. Therefore, the dimensionality reduction of features is one of the most common methods of data preprocessing (Orsenigo and Vercellis, 2013) and its purpose is to reduce the training time of the algorithm and improve the accuracy of final results (Bennasar et al., 2015). In recent years, the research of gene selection methods based on mutual information has received wide attention from scholars. Best individual gene selection (BIF) (Chandrashekar and Sahin, 2014) is the simplest and fastest filtering gene selection algorithm, especially suitable for high-dimensional data.

Battiti utilized the mutual information (MI) between features and class labels [*I*(*f*_*i*_; *c*)] to measure the relevance and the mutual information between features [*I*(*f*_*i*_; *f*_*s*_)] to measure the redundancy (Battiti, 1994). He proposed the Mutual Information Gene selection (MIFS) criterion and it is defined as:

(8) $$\begin{array}{l} J_{MIFS}\left(f_{i}\right)=I\left(f_{i};c\right)-\beta \underset{f_{s}\in \Omega_{S}}{\sum }I\left(f_{i};f_{s}\right),\;f_{i}\in F-\Omega_{S} \end{array}$$

where F is the original feature set, Ω_*S*_ is the selected feature subset, F − Ω_*S*_ is the candidate feature subset and *c* is the class label. β is a configurable parameter to determine the trade–off between relevance and redundancy. However, β is set experimentally, which results in an unstable performance.

Peng et al. (2005) proposed the Minimum-Redundancy Maximum-Relevance (MRMR) criterion and its evaluation function is defined as:

(9) $$\begin{array}{l} J_{mRMR}\left(f_{i}\right)=I\left(f_{i};c\right)-\frac{1}{\left|n_{s}\right|}\underset{f_{s}\in \Omega_{S}}{\sum }I\left(f_{i};f_{s}\right),\;f_{i}\in F-\Omega_{S} \end{array}$$

where |*n*_*s*_| is the number of selected features.

Similarly, other gene selection methods that consider relevance between features and the class label and redundancy between features are concluded, such as Normalized Mutual Information Gene selection (NMIFS) and Conditional Mutual Information (CMI), and they were proposed by Estévez et al. (2009) and Liang et al. (2019) respectively. Their evaluation function are defined as follows:

(10) $$\begin{array}{l} J_{NMIFS}\left(f_{i}\right)=I\left(f_{i};c\right)-\frac{1}{\left|n_{s}\right|}\underset{f_{s}\in \Omega_{S}}{\sum }\frac{I\left(f_{i};f_{s}\right)}{\min\left(H\left(f_{i}\right),H\left(f_{s}\right)\right)},\;f_{i}\in F-\Omega_{S} \end{array}$$

(11) $$\begin{array}{l} J_{CMI}\left(f_{i}\right)=I\left(f_{i};c\right)-\frac{H\left(f_{i}|c\right)}{H\left(f_{i}\right)}\underset{f_{s}\in \Omega_{S}}{\sum }\frac{I\left(f_{s};c\right)I\left(f_{i};f_{s}\right)}{H\left(f_{s}\right)H\left(c\right)},\;f_{i}\in F-\Omega_{S} \end{array}$$

where *H*(*f*_*i*_) is the information entropy and *H*(*f*_*i*_|*c*) is the conditional entropy.

Many gene selection algorithms based on information theory tend to use mutual information as a measure of relevance, which will bring a disadvantage that mutual information tends to select features with more discrete values (Foithong et al., 2012). Thus, the symmetrical uncertainty (Witten and Frank, 2002) (a normalized form of mutual information, *SU*) is adopted to solve this problem. The symmetrical uncertainty can be described as:

(12) $$\begin{array}{l} SU\left(f_{i};c\right)=\frac{2I\left(f_{i};c\right)}{H\left(f_{i}\right)+H\left(c\right)} \end{array}$$

The *SU* can redress the bias of mutual information as much as possible and scale its values to [0,1] by penalizing inputs with large entropies. It will make the performance of gene selection better. Same as MI, for any two features *f*_*i*1_ and *f*_*i*2_, if *SU*(*f*_*i*1_; *c*) > *SU*(*f*_*i*2_; *c*), due to more information can be provided by the former, *f*_*i*1_ and *c* are more relevant. If *SU*(*f*_*i*1_; *f*_*s*_) > *SU*(*f*_*i*2_; *f*_*s*_), owing to the information shared by *f*_*i*1_ and *f*_*s*_ being more and providing less information, *f*_*i*1_ and *f*_*s*_ have greater redundancy.

Additionally, these gene selection algorithms mentioned above fail to take the feature interaction into consideration. After relevance and redundancy analysis, one feature deemed useless may interact with other features to provide more useful information. Especially in complicated biology systems, molecules interacting with each other, they work together to express physiological and pathological changes. If we only consider relevance and redundancy but ignore the feature interaction in data analysis, we may miss some useful features and affect the analysis results (Chen et al., 2015).

Sun et al. (2013), Zeng et al. (2015), and Gu et al. (2020), respectively proposed a gene selection method using dynamic feature weights: Dynamic Weighting-based Gene selection algorithm (DWFS), Interaction Weight based Gene selection algorithm (IWFS) and Redundancy Analysis and Interaction Weight-based gene selection algorithm (RAIW). All of them employ the symmetric uncertainty to measure the relevance between features and the class label, and exploit the three-dimensional interaction information (mentioned at **Information Theory Definition 4**) to measure the interaction between two features and the class label. The evaluation functions are defined as follow:

(13) $$\begin{array}{l} J_{DWFS}\left(f_{i}\right)=SU\left(f_{i};c\right)\times w_{DWFS}\left(f_{i}\right),\;f_{i}\in -\Omega_{S} \end{array}$$

(14) $$\begin{array}{l} J_{IWFS}\left(f_{i}\right)=w_{IWFS}\left(f_{i}\right)\times \left[1+SU\left(f_{i};c\right)\right],\;f_{i}\in F-\Omega_{S} \end{array}$$

(15) $$\begin{array}{l} J_{RAIW}\left(f_{i}\right)=SU\left(f_{i};c\right)\times \left[1-\alpha SU\left(f_{i};f_{s}\right)\right]\\ \;\times w_{RAIW}\left(f_{i}\right),f_{i}\epsilon F-\Omega_{s} \end{array}$$

where *w*(*f*_*i*_) is the weight of each feature and its initial value is set to 1, α is a redundancy coefficient and the value is relevant to the number of dataset's features, *f*_*s*_ is one of features in the selected feature subset. In each round, the feature weight *w*(*f*_*i*_) is updated by their interaction weight factors.

(16) $$\begin{array}{l} \begin{array}{c} w_{DWFS}\left(f_{i}\right)=w_{DWFS}\left({f_{i}}^{\prime }\right)\times \left[1+CR\left(f_{i},f_{s}\right)\right]=w_{DWFS}\left({f_{i}}^{\prime }\right)\\ \;\times \left[1+2\frac{I\left(f_{i};c|f_{s}\right)-I\left(f_{i};c\right)}{H\left(f_{i}\right)+H\left(c\right)}\right]\\ \;=w_{DWFS}\left(f_{i}^{{\;}^{\prime }}\right)\times \left[1+2\frac{I\left(f_{i};f_{s};c\right)}{H\left(f_{i}\right)+H\left(c\right)}\right] \end{array} \end{array}$$

(17) $$\begin{array}{l} w_{IWFS}\left(f_{i}\right)=w_{IWFS}\left(f_{i}^{{\;}^{\prime }}\right)\times IW\left(f_{i},f_{s}\right)\\ =w_{IWFS}\left({f_{i}}^{\prime }\right)\times \left[1+\frac{I\left(f_{i};f_{s};c\right)}{H\left(f_{i}\right)+H\left(f_{s}\right)}\right] \end{array}$$

(18) $$\begin{array}{l} w_{RAIW}\left(f_{i}\right)=w_{RAIW}\left(f_{i}^{{\;}^{\prime }}\right)\times \left[1+If\left(f_{i},f_{s},c\right)\right]\\ \;=w_{RAIW}\left({f_{i}}^{\prime }\right)\times \left[1+\frac{2I\left(f_{i};f_{s};c\right)}{H\left(f_{i}\right)+H\left(f_{s}\right)+H\left(c\right)}\right] \end{array}$$

where $w\left({f_{i}}^{\prime }\right)$ denotes the feature weight of the previous round, *I*(*f*_*i*_; *c*|*f*_*s*_) is the conditional mutual information of *f*_*i*_ and *c* when *f*_*s*_ is given. *I*(*f*_*i*_; *f*_*s*_; *c*) is three-dimensional interaction information. However, we can find that although DWFS and IWFS take into account relevance and interaction, they ignore the redundancy between features. Correlation, redundancy and interaction are all taken into account by RAIW, but there is a no reasonable value for α in a specific dataset.

Furthermore, some other gene selection methods about three-way mutual information are listed and their evaluation function are defined as follows, such as Composition of Feature Relevance (CFR) (Gao et al., 2018a), Joint Mutual Information Maximization (JMIM) (Bennasar et al., 2015), Dynamic Change of Selected Feature with the class (DCSF) (Gao et al., 2018b) and Max-Relevance and Max-Independence (MRI) (Wang et al., 2017).


(19) $$J_{CFR}(f_{i})=\sum_{f_{s}\in \Omega_{S}}I(f_{i};c|f_{s})+\sum_{f_{s}\in \Omega_{S}}I(f_{i};f_{s};c),\;f_{i}\in F-\Omega_{S}$$

(20) $$J_{JMIM}(f_{i})\;=\max[\underset{f_{s}\in \Omega_{S}}{\min}(I(f_{i},f_{s};c))],\;f_{i}\in F-\Omega_{S}$$

(21) $$\begin{array}{l} J_{DCSF}(f_{i})=\sum_{f_{s}\in \Omega_{S}}I(f_{i};c|f_{s})+\sum_{f_{s}\in \Omega_{S}}I(f_{s};c|f_{i})\\ \;-\sum_{f_{s}\in \Omega_{S}}I(f_{i};f_{s}),\;f_{i}\in F-\Omega_{S} \end{array}$$

(22) $$\begin{array}{l} J_{MRI}(f_{i})=I(f_{i};c)+\sum_{f_{s}\in \Omega_{S}}I(f_{i};c|f_{s})\\ \;+\sum_{f_{s}\in \Omega_{S}}I(f_{s};c|f_{i}),\;f_{i}\in F-\Omega_{S} \end{array}$$

where *I*(*f*_*i*_, *f*_*s*_; *c*) is the joint mutual information of *f*_*i*_, *f*_*s*_ and *c*. *I*(*f*_*s*_; *c*|*f*_*i*_) is the conditional mutual information of *f*_*s*_ and *c* when *f*_*i*_ is given. However, these algorithms only take into account three-way mutual information among features and the class label, and none of them considers relevance, redundancy and three-dimensional mutual information between features at the same time, which will affect the performance of these algorithms.

## The Proposed Method-CRIA

In section CNVs Dataset, we firstly introduce the collection of datasets and the process of data processing specifically. Subsequently, we redress other methods' shortcomings and propose an improved gene selection algorithm called CRIA in section The Proposed Algorithm and give it a specific implementation in section Algorithm Implementation. Finally, in section Verify the Performance of CRIA, we verify the performance of CRIA by comparing the experimental results of CRIA and other 8 algorithms on 5 datasets.

### CNVs Dataset

The datasets of copy number variations in different cancer types used in this paper comes from the cBioPortal for Cancer Genomics (http://cbio.mskcc.org/cancergenomics/pancan_tcga/, Release 2/4/2013) (Cerami et al., 2012; Ciriello et al., 2013; Gao et al., 2013). The copy number values in the dataset are generated by Affymetrix SNP 6.0 arrays for the set of samples in the cancer genome atlas (TCGA) study (Liang et al., 2020). The preprocessing analysis of the dataset is performed with GISTIC (Beroukhim et al., 2007). There are 11 cancer types in the cBioPortal database with the largest sample number was 847 and the smallest sample was 135. In order to avoid affecting the experimental results due to the large difference in the number of samples of cancer types, we only select six cancer types with more than 400 samples as our experimental data. The details of six cancer types are listed in Table 1, and totally there are 3480 samples in our experimental dataset.

**Table 1:** The number of samples for each cancer type in this dataset.

**Class label**	**Histology**	**Samples**	**Percentage**
1	UCEC (Uterine corpus endometrial carcinoma)	443	12.73%
2	KIRC (Kidney renal clear cell carcinoma)	490	14.08%
3	OV (Ovarian serous cystadenocarcinoma)	562	16.15%
4	GBM (Glioblastoma multiforme)	563	16.18%
5	COAD/READ (Colon adenocarcinoma/Rect-um adenocarcinoma)	575	16.52%
6	BRCA (Breast invasive carcinoma)	847	24.34%
Total		3,480	100%

In this dataset, each sample consists of labels for 24174 genetic cytobands. The CNV spectrum is divided into five regions/labels by setting four thresholds in cancer algorithm (Mermel et al., 2011). Then, the CNV values are discretized into 5 different values—“-2,” “-1,” “0,” “1,” “2,” where “-2” denotes the deletion of both copies (possibly homozygous deletion), “−1” means the deletion of one copy (possibly heterozygous deletion), “0” corresponds to exactly two copies, i.e., no gain/loss (diploid), “1” denotes a low-level copy number gain and “2” means a high-level copy number amplification (Ciriello et al., 2013).

The CNVs values are preprocessed to the range of [−1,1] with Equation (23).

(23) $$\begin{array}{l} val^{\prime }=\frac{val}{{\left|val\right|}_{\max}} \end{array}$$

where *val* is the value of gene copy number variations of each sample, |*val*|_max_ is the maximum absolute value of gene CNVs among samples and *val*′ is the recalculated value.

### The Proposed Algorithm

In section Related Work, we analyze the 11 gene selection methods and point out their shortcomings. In view of the defects of these algorithms, we propose an improved gene selection algorithm to redress their shortcomings: Correlation-Redundancy and Interaction Analysis based gene selection algorithm (CRIA). This method uses the symmetric uncertainty (*SU*) to measure the correlation between features and the class label and the redundancy among features. In addition, copula entropy is introduced to measure the feature interaction information. Different from the three-way interaction of DWFS, IWFS and RAIW, the proposed algorithm considers the interaction between the candidate feature and the entire set of selected features, instead of being limited to the three-dimensional interaction.

As we know, Shannon's definition of mutual information aims at a pair of random variables, and it measures the correlation between two random variables. Therefore, naturally, many researchers have tried to study how to extend the definition of mutual information from two variables to multivariate situations. In 2011, Ma and Sun published a paper (Ma and Sun, 2011), which contributed to the entropy of information theory. They defined a new concept of entropy in that paper, called Copula Entropy. Copula Entropy is defined on a set of random variables and conformed to symmetry. Therefore, it is a multivariate extension of mutual information, which can be utilized to measure the full-order, non-linear correlation among random variables. They proved the equivalence between copula entropy and the concept of mutual information, which was, mutual information was equal to negative copula entropy (Ma and Sun, 2011).

The copula entropy of $\overset{\to }{x}=\left(x_{1},x_{2},...,x_{N}\right)\in R^{N}$ is defined as:

(24) $$\begin{array}{l} H_{c}\left(\overset{\to }{x}\right)=-\int c\left(\overset{\to }{u}\right)\log c\left(\overset{\to }{u}\right)d\overset{\to }{u} \end{array}$$

where $\overset{\to }{x}$ are random variables with marginal functions $\overset{\to }{u}=\left[F_{1},F_{2},...,F_{N}\right]$ and copula density $c\left(\overset{\to }{u}\right)=\frac{d^{N}C\left(\overset{\to }{u}\right)}{du_{1}du_{2}...du_{N}}$.

Thus, we can use interaction factor *IF*_*CRIA*_, which is defined in Equation (25) to measure the interaction between the candidate feature and the selected feature subset. The meaning of *IF*_*CRIA*_ is that, after adding a random candidate feature *f*_*i*_ into the selected feature subset Ω_*S*_, the amount of interaction information increased relative to the original selected feature subset. So, the bigger value of *IF*_*CRIA*_, the bigger value of interaction between *f*_*i*_ and Ω_*S*_. In each round of calculation, we are supposed to choose the variable that maximizes the *IF*_*CRIA*_ value.

(25) $$\begin{array}{l} IF_{CRIA}=\frac{H_{c}\left(\Omega_{S},f_{i},c\right)}{H_{c}\left(\Omega_{S},c\right)} \end{array}$$

Where *c* is the target class label.

Integrating the correlation between the features and the class label and the redundancy between features that we improved, and the interaction factor we proposed, we can define the evaluation criterion of a candidate feature as follows:

(26) $$\begin{array}{l} \begin{array}{c} J_{CRIA}\left(f_{i}\right)=\underset{f_{i}\in F-\Omega_{S}}{\max}\left\{\left[SU\left(f_{i},c\right)-\frac{1}{n_{s}}\underset{f_{s}\in \Omega_{S}}{\sum }SU\left(f_{i},f_{s}\right)\right]\times IF_{CRIA}\right\}\\ =\underset{f_{i}\in F-\Omega_{S}}{\max}\left\{\left[SU\left(f_{i},c\right)-\frac{1}{n_{s}}\underset{f_{s}\in \Omega_{S}}{\sum }SU\left(f_{i},f_{s}\right)\right]\times \frac{H_{c}\left(\Omega_{S},f_{i},c\right)}{H_{c}\left(\Omega_{S},c\right)}\right\} \end{array} \end{array}$$

For the equation (26), we can see that the proposed algorithm can take into account the relevance between the candidate feature and the class label, redundancy and multi-dimensional interaction among the candidate feature and the selected features at the same time. The formula *SU*(*f*_*i*_, *c*) can denote the relevance and $\frac{1}{n_{s}}\underset{f_{s}\in \Omega_{S}}{\sum }SU\left(f_{i},f_{s}\right)$ calculates the redundancy. Also, the formula $\frac{H_{c}\left(\Omega_{S},f_{i},c\right)}{H_{c}\left(\Omega_{S},c\right)}$ denotes the interaction among the features.

According to the definition of copula entropy and Equation (24), there is a theorem.

**Theorem 1:** The mutual information of random variables is equivalent to their negative copula entropy (Ma and Sun, 2011):

(27) $$\begin{array}{l} I\left(\overset{\to }{x}\right)=-H_{c}\left(\overset{\to }{x}\right) \end{array}$$

According to Theorem 1, the value of copula entropy can be calculated by the MI of multivariates. The definition of mutual information extended from two variables to multivariate is described as follows:

(28) $$\begin{array}{l} \begin{array}{c} I\left(X_{m},c\right)=\iint p\left(X_{m},c\right)\log\frac{p\left(X_{m},c\right)}{p\left(X_{m}\right)p\left(c\right)}dX_{m}dc\\ =\iint p\left(X_{m-1},x_{m},c\right)\log\frac{p\left(X_{m-1},x_{m},c\right)}{p\left(X_{m-1},x_{m}\right)p\left(c\right)}dX_{m-1}dx_{m}dc\\ =\int \ldots \int p\left(x_{1},\ldots ,,x_{m},c\right)\log\frac{p\left(x_{1},\ldots ,x_{m},c\right)}{p\left(x_{1},\ldots ,x_{m}\right)p\left(c\right)}dx_{1},\ldots ,dx_{m}dc \end{array} \end{array}$$

where *X*_*m*_ = {*x*_1_, *x*_2_, …, *x*_*m*−1_, *x*_*m*_} = {*X*_*m*−1_, *x*_*m*_}.

According to the Equation (28), we have:

(29) $$\begin{array}{l} \begin{array}{c} H\left(X_{m-1},x_{m}\right)=H\left(X_{m}\right)=\overset{m}{\underset{i=1}{\sum }}H\left(x_{i}\right)-I\left(X_{m}\right)\\ H\left(X_{m-1},x_{m},c\right)=H\left(X_{m},c\right)=H\left(c\right)+\overset{m}{\underset{i=1}{\sum }}H\left(x_{i}\right)-I\left(X_{m},c\right) \end{array} \end{array}$$

Therefore,

(30) $$\begin{array}{l} \begin{array}{c} I\left(X_{m},c\right)=H\left(x_{1}\right)+\ldots +H\left(x_{m}\right)+H\left(c\right)-H\left(x_{1},\ldots ,x_{m},c\right)\\ I\left(X_{m},{\hat{X}}_{s},c\right)=H\left(x_{1}\right)+\ldots +H\left(x_{m}\right)+H\left({\hat{X}}_{s}\right)+H\left(c\right)\\ -H\left(x_{1},\ldots ,x_{m},{\hat{X}}_{s},c\right) \end{array} \end{array}$$

According to Equation (26), (27) and (30), we have:

(31) $$\begin{array}{l} \begin{array}{c} J_{CRIA}\left(f_{i}\right)=\underset{f_{i}\in F-\Omega_{S}}{\max}\left\{\left[SU\left(f_{i},c\right)-\frac{1}{n_{s}}\underset{f_{s}\in \Omega_{S}}{\sum }SU\left(f_{i},f_{s}\right)\right]\times IF_{CRIA}\right\}\\ =\underset{f_{i}\in F-\Omega_{S}}{\max}\left\{\left[SU\left(f_{i},c\right)-\frac{1}{n_{s}}\underset{f_{s}\in \Omega_{S}}{\sum }SU\left(f_{i},f_{s}\right)\right]\times \frac{I\left(\Omega_{S},f_{i},c\right)}{I\left(\Omega_{S},c\right)}\right\} \end{array} \end{array}$$

Let Ω_*S*_ = {*f*_1_, *f*_2_, …, *f*_*m*_}, Since,

(32) $$\begin{array}{l} \frac{I\left(\Omega_{S},f_{i},c\right)}{I\left(\Omega_{S},c\right)}=\frac{\overset{m}{\underset{k=1}{\sum }}H\left(f_{k}\right)+H\left(f_{i}\right)+H\left(c\right)-H\left(\Omega_{S},f_{i},c\right)}{\overset{m}{\underset{k=1}{\sum }}H\left(f_{k}\right)+H\left(c\right)-H\left(\Omega_{S},c\right)} \end{array}$$

Therefore,

(33) $$\begin{array}{l} \begin{array}{c} J_{CRIA}\left(f_{i}\right)=\underset{f_{i}\in F-\Omega_{S}}{\max}\{\left[SU\left(f_{i},c\right)-\frac{1}{n_{s}}\underset{f_{s}\in \Omega_{S}}{\sum }SU\left(f_{i},f_{s}\right)\right]\times \\ \frac{\overset{m}{\underset{k=1}{\sum }}H\left(f_{k}\right)+H\left(f_{i}\right)+H\left(c\right)-H\left(\Omega_{S},f_{i},c\right)}{\overset{m}{\underset{k=1}{\sum }}H\left(f_{k}\right)+H\left(c\right)-H\left(\Omega_{S},c\right)}\} \end{array} \end{array}$$

**Figure 1:**
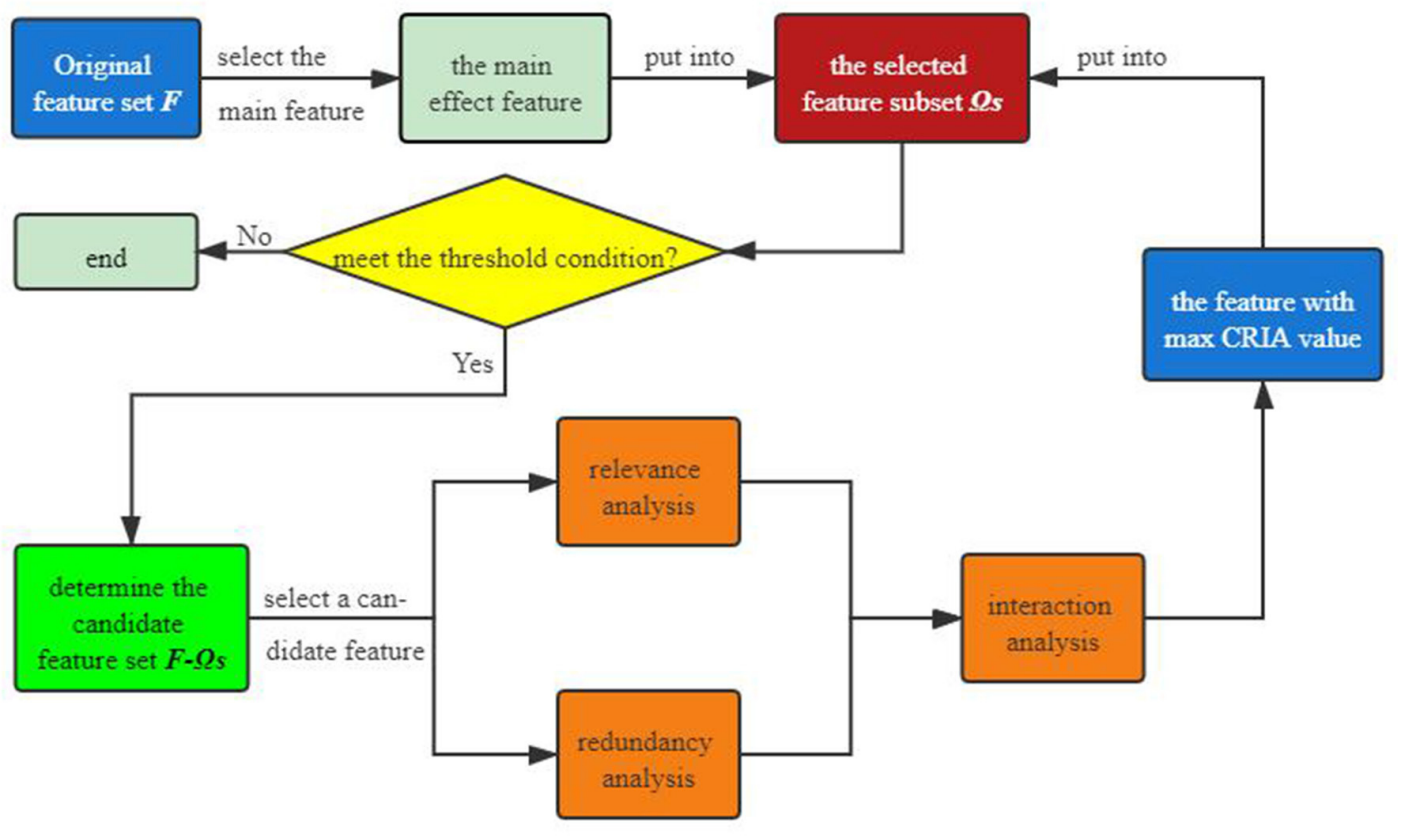
General flow chart of the proposed algorithm.

The general flow chart of the proposed algorithm is presented in Figure 1 we can see that an original feature set F is first given, from which we select the main effect feature that maximizes the value of (12). Then the main feature is put into the selected feature subset Ω_*S*_. For each feature in the candidate feature set, after conducting correlation and redundancy analysis, we are next supposed to use (25) to perform interaction analysis on it. Choose the feature that maximizes the value of (33), which then is put into the selected feature set. If the number of the selected features meets the threshold condition, the above steps will be executed again, otherwise the program ends directly.

### Algorithm Implementation

We propose a gene selection method based on correlation-redundancy and interaction analysis. The pseudo code of CRIA algorithm is described as follows.

Here, for the CNVs dataset, we set the value of the threshold *M* to be 200 to reduce the calculation time and avoid curse of dimensionality. In addition, we need to control the number of selected features to be same as the method proposed by Zhang et al. (2016).

The CRIA algorithm consists of two stages:

Stage 1 (lines 1–7): In this part, the selected feature subset Ω_*S*_ and the original feature set F are first initialized. For each feature in the original feature set *f*_*i*_, the symmetrical uncertainty *SU*(*f*_*i*_; *c*) between *f*_*i*_ and class label *c* is calculated. The feature whose value of symmetrical uncertainty with class label is the maximum is selected out and added into the selected features subset Ω_*S*_, which we name “the main effect feature.”

Stage 2 (lines 8-18): The second stage mainly calculates the correlation measure *SU*(*f*_*i*_; *c*) and the redundancy measure $\frac{1}{n_{s}}\underset{f_{s}\in \Omega_{S}}{\sum }SU\left(f_{i},f_{s}\right)$. Then the interaction value *IF*_*CRIA*_ between Ω_*S*_, *f*_*i*_ and *c* is updated. *J*_*CRIA*_(*f*_*i*_) is calculated and the feature with the maximum value is added into the selected feature subset Ω_*S*_. This procedure terminates until the number of selected features is no less than predefined threshold *M*.

According to Algorithm 1, when the size of the feature subset reaches the set threshold *M*, the procedure will be terminated. The value of the threshold setting should be determined by different datasets. A small *M* can reduce the amount of calculation but may also lose many effective features that are useful; a large *M* will increase the amount of calculation but may improve the accuracy of final result (Foithong et al., 2012). Actually, when the threshold exceeds a certain value, the accuracy of the final result will not only not increase, but may decrease, and it will bring computational complexity. The selected features are ranked according to the value of the evaluation function *J*_*CRIA*_(*f*_*i*_) from largest to smallest.

**Algorithm 1:** CRIA: correlation-redundancy and interaction analysis based gene selection algorithm.

**Input** *N*: the number of original features, *M*: the number of features to be selected, *n*_*s*_: the number of selected features.
**Output**: the selected feature subset (Ω_*S*_ ⊆ *F*).
1 First initializes Ω_*S*_ = ∅, *F* = {*f*_1_, *f*_2_, …, *f*_*N*_};
2 for each *f*_*i*_ ∈ *F* do:
3 calculate *SU*(*f*_*i*_, *c*);
4 end for
5 select the feature *f*_*i* max_ ∈ 𝔽 with the largest value of *SU*(*f*_*i*_, *c*);
6 Ω_*S*_ = Ω_*S*_ ∪ {*f*_*i* max_};
7 *F* = *F* − {*f*_*i* max_};
8 while *n*_*s*_ ≤ *M* do:
9 for *f*_*i*_ ∈ 𝔽 do:
10 calculate $SU\left(f_{i},c\right)-\frac{1}{n_{s}}\underset{f_{s}\in \Omega_{S}}{\sum }SU\left(f_{i},f_{s}\right)$;
11 calculate $IF_{CRIA}=\frac{H_{c}\left(\Omega_{S},f_{i},c\right)}{H_{c}\left(\Omega_{S},c\right)}$;
12 calculate $J_{CRIA}\left(f_{i}\right)=\left[SU\left(f_{i},c\right)-\frac{1}{n_{s}}\underset{f_{s}\in \Omega_{S}}{\sum }SU\left(f_{i},f_{s}\right)\right]\times IF_{CRIA}$ and append it into a list;
13 end for
14 select the feature *f*_*k* max_ ∈ 𝔽 with the largest value of *J*_*CRIA*_(*f*_*i*_) from list;
15 Ω_*S*_ = Ω_*S*_ ∪ {*f*_*k* max_};
16 *F* = *F* − {*f*_*k* max_};
17 end while
18 output Ω_*S*_.

### Verify the Performance of CRIA

Eight gene selection algorithms—JMIM (Bennasar et al., 2015), mRMR (Peng et al., 2005), DWFS (Sun et al., 2013), IWFS (Zeng et al., 2015), RAIW (Gu et al., 2020), CFR (Gao et al., 2018a), DCSF (Gao et al., 2018b), and MRI (Wang et al., 2017) are used to compare with CRIA to examine the performance of our proposed method.

The datasets used in validation experiment come from Arizona State University (ASU) datasets (Li et al., 2017), which include four biological data and one other type of data (digit recognition). They are all high-dimensional data. The smallest feature number is 2000 and the largest feature number is 9182 among them. The specific details of these datasets are shown in Table 2. We only use minimum description length method (Fayyad and Irani, 1993) for gene selection and utilize it to convert these numerical features.

**Table 2:** Datasets for comparison between CRIA algorithm and other algorithms.

**Datasets type**	**No**.	**Datasets**	**Samples**	**Features**	**Classes**	**Types**
Biological data	1	leukemia	72	7,070	2	Discrete
	2	Carcinoma	174	9,182	11	Continuous
	3	colon	62	2,000	2	Discrete
	4	TOX_171	171	5,748	4	Continuous
Digit recognition	5	Gisette	7,000	5,000	2	Continuous

The number of features *N* used in the experiment is reduced to 50 and three classifiers—IB1, J48 and Naïve Bayes are exploited. The parameters of the classifiers are set to the default parameters of Waikato Environment for Knowledge Analysis (WEKA) (Hall et al., 2009). We use 10 times of ten-fold cross-validation to avoid the influence of randomness on experimental results. Then mean value and Standard Deviation (STD) are taken as the comparison indices of performance of each algorithm and STD is defined as follows:

(34) $$\begin{array}{l} STD=\sqrt{\frac{1}{n_{run}}\overset{N}{\underset{i=1}{\sum }}\left(ACC_{i}-u\right)} \end{array}$$

where *n*_*run*_ is the number of times of our experiments, here we set *n*_*run*_ = 10, *ACC* is the classification accuracy, *u* represents the average value of *ACC*, and *N* denotes the number of samples. The bigger *ACC*, the better performance, and the smaller *STD*, the higher stability.

The comparison results between the proposed algorithm and other gene selection algorithms are shown in Tables 3–5. As shown in Table 3, for the five data sets in the experiment, we can see that the classification results of CRIA in four data sets are better than other eight algorithms, which ranking first, except TOX_171, ranking fourth. Compared with other algorithms, the average accuracy of CRIA is increased by 2.42–5.08%. In Table 4, CRIA also outperforms the other 8 algorithms on four data sets except TOX_171, on which the experimental results of CRIA ranking third. The biggest improved rate of the proposed algorithm is 10.05% and the smallest one is 3.73%. From Table 5, we can find that the results of CRIA on the three data sets are superior to other algorithms, ranking first. However, on the datasets of Carcinoma and TOX_171, compared with the maximum values, the experimental accuracies of CRIA are slightly decreased by 0.50 and 1.71%, ranking second and third respectively. From the perspective of average accuracy, CRIA's result is better than other algorithms, and it is improved by 2.11–8.67%.

**Table 3:** Comparision (mean ± std.dev.) of performance between CRIA and other 8 algorithms with J48 classifier.

**Datasets**	**CRIA** **(proposed)**	**RAIW**	**mRMR**	**DWFS**	**IWFS**	**JMIM**	**MRI**	**CFR**	**DCFS**
leukemia	**95.00 ± 1.17** **(1)**	93.08 ± 0.67 (4)	93.09 ± 0.64 (3)	92.51 ± 1.14 (7)	92.05 ± 0.73 (8)	93.36 ± 0.59 (2)	92.70 ± 0.98 (6)	93.07 ± 1.10 (5)	91.50 ± 1.44 (9)
Carcinoma	**75.17 ± 1.67** **(1)**	71.73 ± 1.80 (3)	71.78 ± 2.26 (2)	69.79 ± 1.58 (4)	64.47 ± 1.82 (9)	64,84 ± 1.68 (5)	68.65 ± 2.20 (6)	68.01 ± 1.97 (7)	65.53 ± 1.89 (8)
colon	**79.68 ± 2.54** **(1)**	76.55 ± 3.86 (5)	77.14 ± 3.31 (4)	74.32 ± 2.48 (8)	76.32 ± 2.07 (6)	77.28 ± 3.77 (3)	77.30 ± 4.73 (2)	74.71 ± 3.96 (7)	73.31 ± 3.77 (9)
TOX_171	62.01 ± 1.61 (4)	62.21 ± 2.04 (2)	56.61 ± 2.21 (8)	60.94 ± 3.08 (5)	62.09 ± 2.14 (3)	57.49 ± 3.01 (9)	59.78 ± 2.02 (7)	60.18 ± 1.91 (6)	**62.26 ± 2.71** **(1)**
gisette	**93.71 ± 0.17** **(1)**	92.40 ± 0.08 (6)	92.02 ± 0.08 (8)	92.66 ± 0.10 (5)	92.05 ± 0.12 (7)	91.18 ± 0.12 (9)	92.84 ± 0.08 (3)	92.82 ± 0.07 (4)	93.35 ± 0.08 (2)
Avg.acc	81.11	79.19	78.53	78.04	77.40	77.63	78.25	77.76	77.19
Avg.rank	1.60	4.00	5.00	5.80	6.60	5.60	4.80	5.80	5.80
Improved rate	–	2.42%	3.29%	3.93%	4.79%	4.48%	3.65%	4.31%	5.08%

*The meaning of the bold values represent the best performance achieved on a certain dataset for the nine methods*.

**Table 4:** Comparision (mean ± std.dev.) of performance between CRIA and other 8 algorithms with IB1 classifier.

**Datasets**	**CRIA** **(proposed)**	**RAIW**	**mRMR**	**DWFS**	**IWFS**	**JMIM**	**MRI**	**CFR**	**DCFS**
leukemia	**99.44 ± 0.97** **(1)**	97.03 ± 0.87 (2)	96.16 ± 0.43 (4)	95.56 ± 0.90 (7)	88.75 ± 1.88 (9)	96.61 ± 0.78 (3)	96.13 ± 0.90 (5)	95.73 ± 0.95 (6)	94.22 ± 0.80 (8)
Carcinoma	**86.84 ± 0.50** **(1)**	82.45 ± 1.33 (2)	81.39 ± 1.02 (4.5)	82.32 ± 1.07 (3)	76.88 ± 1.97 (9)	81.10 ± 1.06 (7)	81.39 ± 1.16 (4.5)	81.35 ± 1.08 (6)	80.96 ± 1.18 (8)
colon	**86.77 ± 1.27** **(1)**	78.60 ± 2.10 (2)	78.22 ± 1.54 (3)	75.94 ± 2.32 (5)	70.87 ± 1.97 (9)	76.69 ± 2.35 (4)	71.77 ± 3.41 (8)	72.24 ± 2.17 (7)	74.87 ± 1.80 (6)
TOX_171	84.56 ± 0.52 (3)	85.13 ± 1.26 (2)	78.14 ± 1.36 (8)	**85.19 ± 1.30** **(1)**	82.59 ± 1.78 (5)	76.68 ± 1.68 (9)	81.69 ± 1.64 (7)	82.05 ± 1.28 (6)	84.04 ± 1.48 (4)
gisette	**93.75 ± 0.14** **(1)**	91.88 ± 0.08 (6)	91.26 ± 0.09 (7)	92.26 ± 0.07 (5)	91.05 ± 0.15 (8)	90.20 ± 0.10 (9)	92.70 ± 0.06 (3)	92.58 ± 0.05 (4)	93.13 ± 0.10 (2)
Avg.acc	90.27	87.02	85.03	86.25	82.03	84.26	84.74	84.79	85.44
Avg.rank	1.40	2.80	5.30	4.20	8.00	6.40	5.50	5.80	5.60
Improved rate	–	3.73%	6.16%	4.66%	10.05%	7.13%	6.53%	6.46%	5.65%

*The meaning of the bold values represent the best performance achieved on a certain dataset for the nine methods*.

**Table 5:** Comparision (mean ± std.dev.) of performance between CRIA and other 8 algorithms with Naïve Bayes classifier.

**Datasets**	**CRIA** **(proposed)**	**RAIW**	**mRMR**	**DWFS**	**IWFS**	**JMIM**	**MRI**	**CFR**	**DCFS**
leukemia	**99.58 ± 0.67** **(1)**	97.44 ± 0.66 (2)	96.27 ± 0.30 (7)	97.03 ± 0.70 (4)	95.48 ± 1.96 (9)	96.18 ± 0.39 (8)	97.15 ± 0.80 (3)	96.79 ± 0.71 (5)	96.70 ± 0.58 (6)
Carcinoma	81.61 ± 1.11 (2)	**82.02 ± 0.83** **(1)**	80.23 ± 1.33 (7)	81.58 ± 0.95 (3)	76.38 ± 1.85 (9)	80.19 ± 1.28 (8)	80.41 ± 0.86 (5)	80.34 ± 0.87 (6)	80.46 ± 1.09 (4)
colon	**88.71 ± 0.00** **(1)**	82.97 ± 1.34 (2)	82.70 ± 1.20 (3)	80.72 ± 1.47 (6)	74.39 ± 4.37 (9)	81.66 ± 1.45 (5)	79.84 ± 2.40 (7)	78.95 ± 1.72 (8)	82.32 ± 2.08 (4)
TOX_171	69.53 ± 0.70 (3)	**70.74 ± 1.07** **(1)**	63.73 ± 1.61 (8)	68.68 ± 1.26 (4)	65.64 ± 1.63 (7)	60.41 ± 2.35 (9)	66.96 ± 1.56 (6)	67.04 ± 1.74 (5)	70.28 ± 1.60 (2)
gisette	**93.16 ± 0.05** **(1)**	90.46 ± 0.13 (2)	88.26 ± 0.02 (4)	87.69 ± 0.08 (5)	86.23 ± 0.24 (8)	86.01 ± 0.05 (9)	87.60 ± 0.04 (6)	87.48 ± 0.03 (7)	89.46 ± 0.05 (3)
Avg.acc	86.52	84.73	82.24	83.14	79.62	80.89	82.39	82.12	83.84
Avg.rank	1.60	1.80	5.80	4.40	8.40	7.80	5.40	6.20	3.80
Improved rate	–	2.11%	5.20%	4.07%	8.67%	6.96%	5.01%	5.36%	3.20%

*The meaning of the bold values represent the best performance achieved on a certain dataset for the nine methods*.

## Results and Discussions

### Evaluation Metrics of Experimental Results

Four evaluation metrics—precision, recall, accuracy and F1-score are utilized to evaluate the performance of the corresponding method and values of these criteria are defined as equation (35).

(35) $$\begin{array}{r} precision=\frac{T_{P}}{T_{P}+F_{P}}\\ recall=\frac{T_{P}}{T_{P}+F_{N}}\\ accuracy=\frac{T_{P}+T_{N}}{T_{P}+T_{N}+F_{P}+F_{N}}\\ F1-score=\frac{2\times precision\times recall}{precision+recall} \end{array}$$

where *T*_*P*_, *T*_*N*_, F_*P*_, and *F*_*N*_ denotes the numbers of true positives, true negatives, false positives, and false negatives respectively.

### The CRIA and IFS Results

As mentioned in section Evaluation Metrics of Experimental Results, each sample is represented by 24,174 features, each of which indicates the expression level of genes. The 24174 feature genes are sorted by CRIA value in descending order. However, we only select the top 200 features in this work for the consideration of computational time and curse of dimensionality. The top 15 key feature genes chosen by CRIA defined by equation (26) are listed in Table 6.

**Table 6:** The top 15 feature genes chosen by CRIA defined as equation (26).

**Ranked order**	**Official name**	**Official full gene name**	**Category**	**CRIA value**
1	RPS15	ribosomal protein S15	Protein Coding	0.168
2	TBC1D5	TBC1 Domain Family Member 5	Protein Coding	0.089
3	CUL2	Cullin 2	Protein Coding	0.093
4	SMPD3	Sphingomyelin Phosphodiesterase 3	Protein Coding	0.089
5	CTAGE10P	CTAGE Family Member 10, Pseudogene	Pseudogene	0.071
6	C1orf98	Chromosome 1 Open Reading Frame 98	Protein Coding	0.043
7	ZNF281	Zinc Finger Protein 281	Protein Coding	0.061
8	CDKN2A	Cyclin Dependent Kinase Inhibitor 2A	Protein Coding	0.161
9	EGFR	Epidermal Growth Factor Receptor	Protein Coding	0.121
10	TMEM98	Transmembrane Protein 98	Protein Coding	0.103
11	CTBP2	C-Terminal Binding Protein 2	Protein Coding	0.083
12	SEMA6A	Semaphorin 6A	Protein Coding	0.081
13	MIR1208	MicroRNA 1208	RNA Gene	0.077
14	RBFOX1	RNA Binding Fox-1 Homolog 1	Protein Coding	0.069
15	CDC25A	Cell Division Cycle 25A	Protein Coding	0.066

We use the Incremental Gene selection (IFS) (Yang et al., 2019) to determine the optimal feature set. The first 200 features are added one by one to a feature subset in order. Each time a feature is added, a classifier is trained and examined. So, 200 classifiers are constructed. We use the criteria of accuracy to evaluate the performance of all the 200 classifiers and then we choose the classifier with the highest accuracy as the final one. The corresponding feature subset that the final classifier used is deemed to be the optimal feature set.

**Figure 2:**
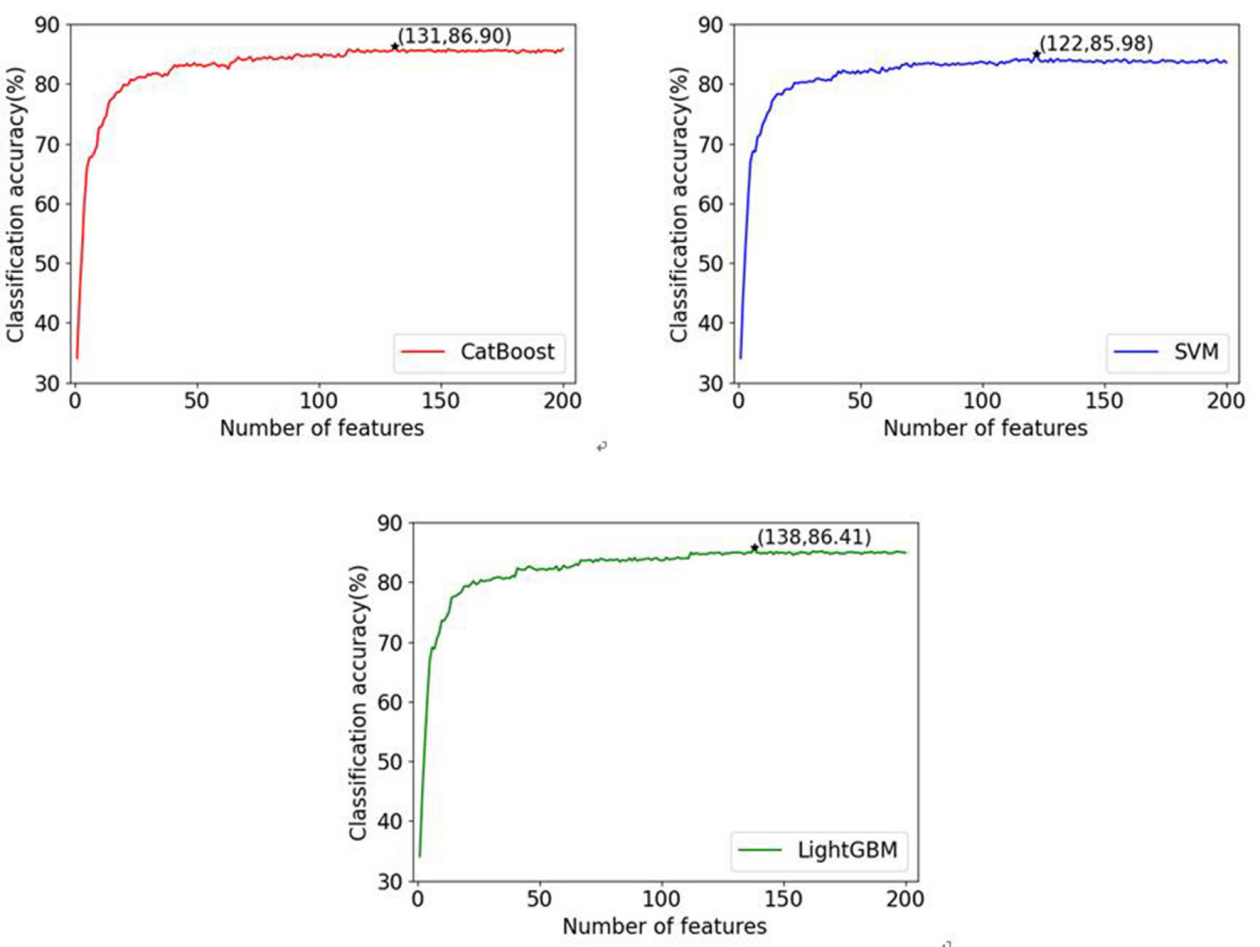
Classification accuracies of three classifiers (CatBoost, LightGBM and SVM) with different numbers of features during the IFS procedure. The top 200 feature genes are selected by CRIA method.

In this paper, three commonly used classifiers are adopted to verify the generalization performance of the proposed gene selection method on different classifiers. ten-fold cross-validation is used to evaluate our algorithm with the selected features. The complete data set is randomly split into 10 parts of approximately equal size. The three classifiers are trained 10 times; nine of the 10 subsets are used as the training datasets, and the remaining one is the test dataset. The average values of accuracy for each classifier are calculated and the IFS results are shown in Figure 2. Here, we name our methods as CRIA_CatBoost, CRIA_SVM and CRIA_LightGBM. From Figure 2, it can be seen that the highest accuracy of 86.90% for CRIA_CatBoost method followed by 86.41% for CRIA_LightGBM and 85.98% for CRIA_SVM method, with only using the CNVs of 131 genes, 138 genes and 122 genes respectively.

### The Proposed Algorithm Performance

For the different classifiers used in this work, after determining the optimal numbers of features according to the CRIA and IFS results, the classification performance can be further analyzed. The average values of three metrics-precision, recall and F1-score defined in Equation (35) on 10 test datasets are listed in Table 7.

**Table 7:** Average performance of precision, recall and F1-score on 10 test datasets with three classifiers *via* ten-fold cross-validation (%).

**Metrics**		**UCEC**	**KIRC**	**OV**	**GBM**	**COAD/** **READ**	**BRCA**
Precision	CRIA_CatBoost	74.31	93.74	84.59	94.63	89.67	84.48
	CRIA_SVM	70.47	90.33	85.40	95.64	88.54	84.76
	CRIA_LightGBM	71.46	93.37	82.84	95.72	90.23	84.71
Recall	CRIA_CatBoost	73.14	91.63	87.90	90.76	86.09	88.67
	CRIA_SVM	73.81	89.59	88.43	89.70	83.30	87.96
	CRIA_LightGBM	72.91	92.04	89.32	91.30	83.48	87.01
F1-score	CRIA_CatBoost	73.72	92.67	86.21	92.65	87.84	86.52
	CRIA_SVM	72.13	89.96	86.89	92.57	85.84	86.33
	CRIA_LightGBM	72.18	92.70	85.96	93.46	86.72	85.84

### Performance Comparison With Other Methods

After selecting important features, we use three common classifiers—CatBoost, SVM and LightGBM to predict cancer samples. The performance of our methods are compared with other two classification methods published before whose experimental dataset is the same as ours. Liang et al. (2020) used a method called CNA_origin which was composed of a stacked autoencoder and an one-dimensional convolutional neural network. The 24,174 gene features were extracted to 100 genes by the autoencoder, and then these 100 gene features were put into the 1D CNN for classification (Liang et al., 2020). A computationally method for cancer types classification proposed by Zhang et al. (2016) was named as mRMR_Dagging here because there was no specific method name given by authors. It first used mRMR and IFS to select 19 of the 24,174 genes as classification features, and then used the Dagging algorithm to give the final results.

In Table 8, it can be seen that if the results of our methods are superior to CNA_origin and mRMR_Dagging, they are marked in bold. Similarly, if the largest of CNA_origin and mRMR_Dagging results is better than our method, it is also marked in bold. Table 8 demonstrated that the performance of our methods is superior to CNA_origin and mRMR_Dagging for UCEC, KIRC, GBM, and COADREAD. For UCEC, the recall and F1-score of our methods (CRIA_Cat- Boost, CRIA_SVM and CRIA_LightGBM) are all superior to CNA_origin and mRMR_Dagging. The best precision of our methods is 0.12 percentage points higher than mRMR_Dagging. SVM and LightGBM are slightly worse than mRMR_Dagging with reductions of 5.28 and 3.82% in precision respectively. For KIRC, the precision and F1-score are all superior to CNA_origin and mRMR_Dagging except the F1-score of SVM, which performs slightly worse than the CNA_origin with reductions of 2.61%. Compared with the best, CNA_origin, the recall of our methods are decreased by 4.77% for CatBoost, 7.15% for SVM and 4.30% for LightGBM. For OV, compared with CNA_origin, the recall of our methods is at least increased by 1.36%. The precision and F1-score are slightly worse than CNA_origin, with reductions at most of 8.40, and 2.37%, respectively. For GBM and COADREAD, our methods are better than CNA_origin and mRMR_Dagging on all evaluation indicators. Compared with the best of the other two algorithms, the worst precision of our methods is increased by 1.64 and 8.53%, respectively, the worst recall is increased by 4.39 and 12.86%, respectively, and the worst F1-score is increased by 4.58 and 10.76%, respectively. For BRCA, the worst among our methods performs slightly worse than the best CNA_origin algorithm, with reductions of 3.57% in precision, 6.09% in recall and 4.66% in F1-score respectively.

**Table 8:** Performance comparison of the proposed algorithm predictions with those of other methods (%).

**Cancer**	**Predictor**	**Precision**	**Recall**	**F1-score**
UCEC	CRIA_CatBoost	**74.31**	**73.14**	**73.72**
	CRIA_SVM	70.47	**73.81**	**72.13**
	CRIA_LightGBM	71.46	**72.91**	**72.18**
	CNA_origin	67.92	72.00	69.90
	mRMR_Dagging	74.19	46.93	57.50
KIRC	CRIA_CatBoost	**93.74**	91.63	**92.67**
	CRIA_SVM	**90.33**	89.59	89.96
	CRIA_LightGBM	**93.37**	92.04	**92.70**
	CNA_origin	88.89	**96.00**	92.31
	mRMR_Dagging	80.85	92.68	86.36
OV	CRIA_CatBoost	84.59	**87.90**	86.21
	CRIA_SVM	85.40	**88.43**	86.89
	CRIA_LightGBM	82.84	**89.32**	85.96
	CNA_origin	**89.80**	86.72	**88.00**
	mRMR_Dagging	84.61	75.86	80.00
GBM	CRIA_CatBoost	**94.63**	**90.76**	**92.65**
	CRIA_SVM	**95.64**	**89.70**	**92.57**
	CRIA_LightGBM	**95.72**	**91.30**	**93.46**
	CNA_origin	93.10	84.38	88.52
	mRMR_Dagging	88.70	85.93	87.30
COADREAD	CRIA_CatBoost	**89.67**	**86.09**	**87.84**
	CRIA_SVM	**88.54**	**83.30**	**85.84**
	CRIA_LightGBM	**90.23**	**83.48**	**86.72**
	CNA_origin	81.58	73.81	77.50
	mRMR_Dagging	60.00	73.46	66.05
BRCA	CRIA_CatBoost	84.48	88.67	86.52
	CRIA_SVM	84.76	87.96	86.33
	CRIA_LightGBM	84.71	87.01	85.84
	CNA_origin	**87.50**	**92.31**	**89.84**
	mRMR_Dagging	79.16	87.35	83.06

**Figure 3:**
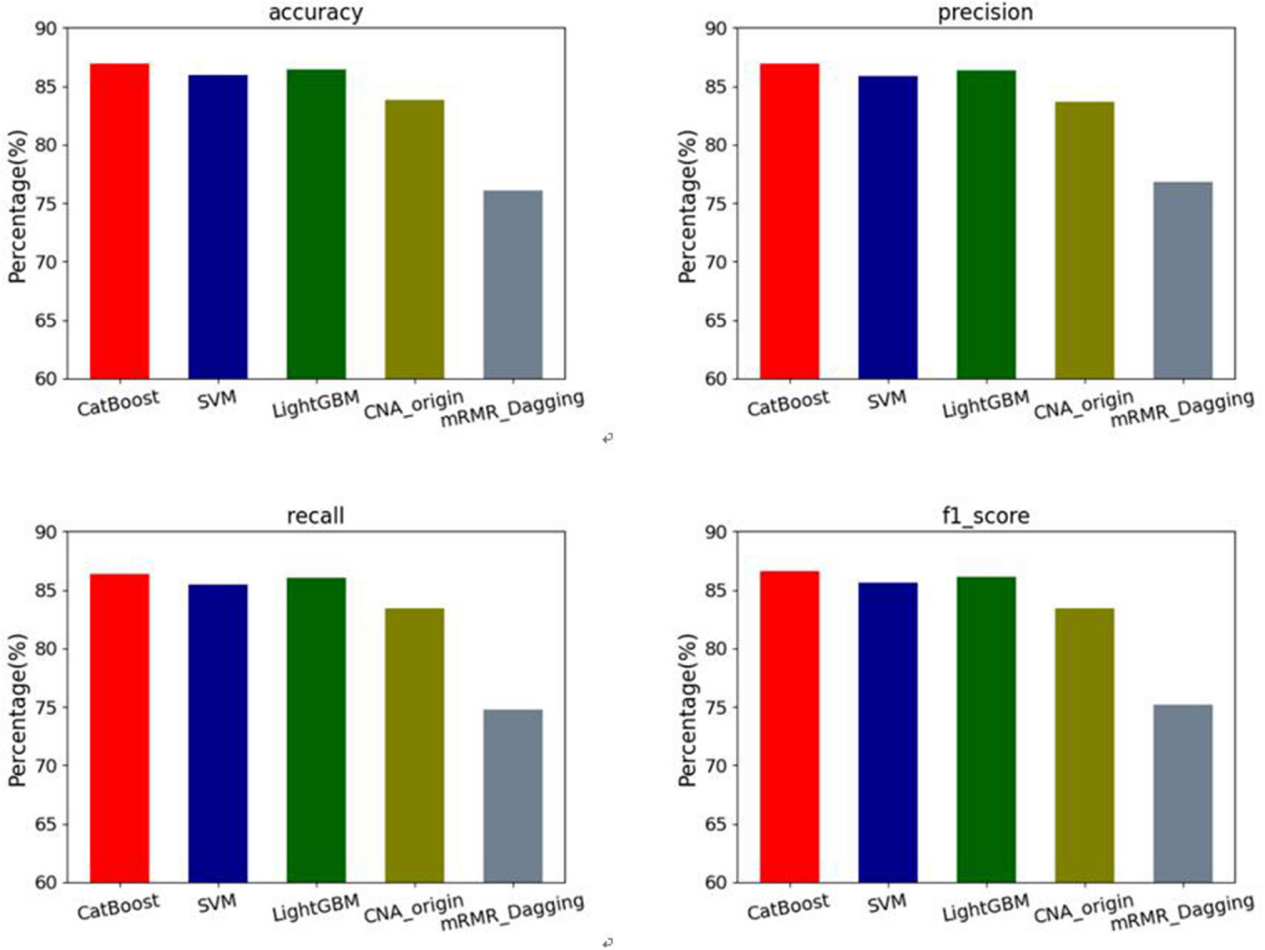
Performance comparison of 4 evaluation metrics: accuracy, precision, recall and F1-score among our methods and the other two algorithms (CNA_origin and mRMR_Dagging).

In addition, the macro-average results of four evaluation metrics: accuracy, precision, recall and F1-score are used to assess our methods and the other two algorithms on the datasets of six types of cancers. The results can be seen in Figure 3. For accuracy, our methods have mean values of 86.90% for CatBoost, 86.41% for LightGBM and 85.98% for SVM respectively, which are increased by 3.69, 3.10, and 2.59% compared with CNA_origin. For precision, the average values of our methods are 86.61, 86.39, and 85.86%, which are increased by 3.49, 3.23, and 2.59%, respectively compared with the best among CNA_origin and mRMR_Dagging. For recall, our methods' mean values are 86.37, 86.01, and 85.47%, which are 2.92, 2.56 and 2.02 percentage points higher than CNA_origin, respectively. For F1-score, compared with our methods, whose average values are 86.60, 86.14, and 85.62%, CNA_origin is decreased by 3.71, 3.19, and 2.60%, respectively.

### Further Discussion

**Figure 4:**
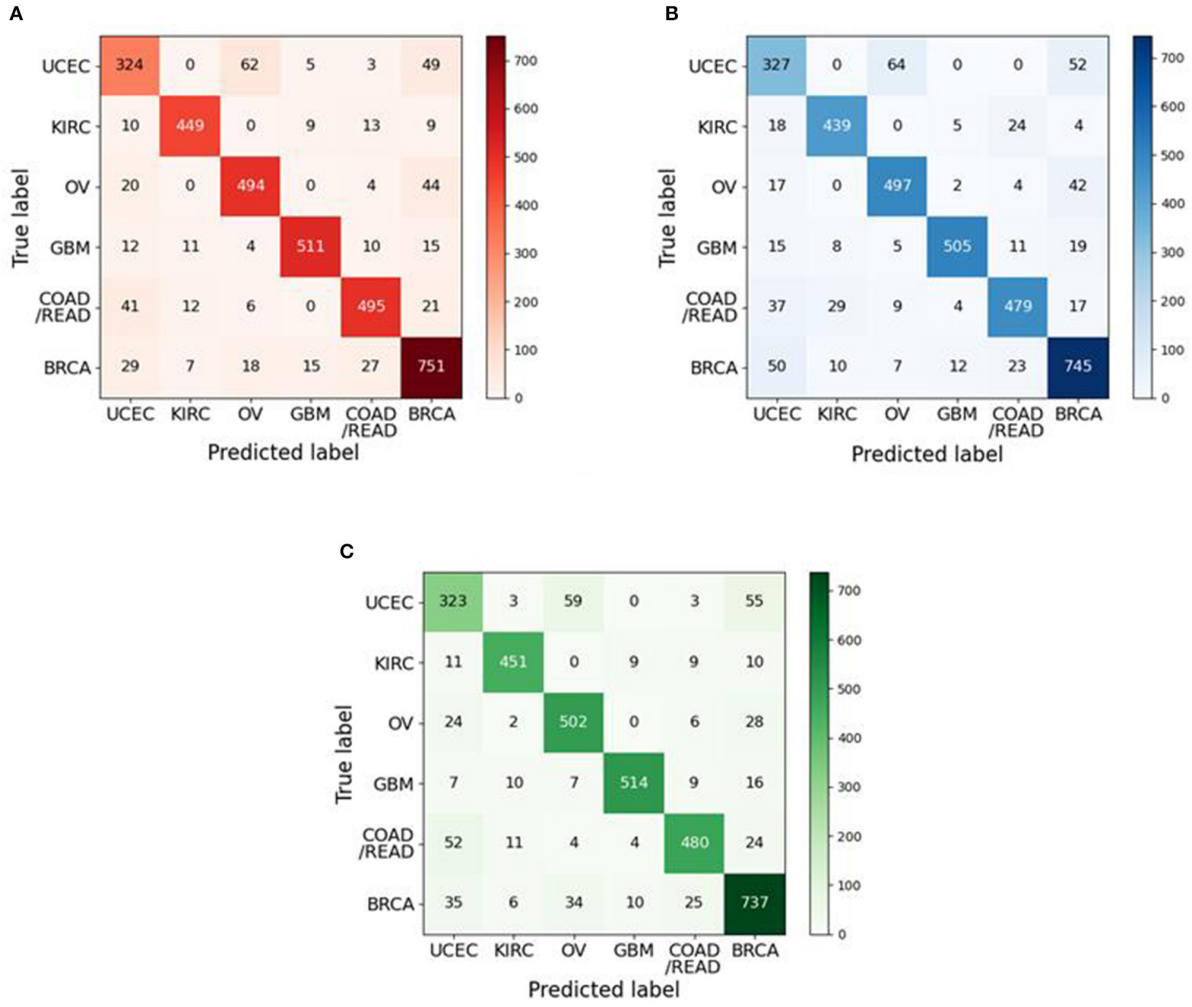
Confusion matrices on the test groups: **(A)** CRIA_CatBoost; **(B)** CRIA_SVM; **(C)** CRIA_LightGBM.

In order to study the relationship between the classes, we also summarize the confusion matrices in Figure 4 for class predictions using our methods. From Figure 4, we can find that there existed a high error rate when predicting the samples of UCEC. Regardless of whether it is CRIA_CatBoost, CRIA_SVM or CRIA_LightGBM, more than 10% of the UCEC samples are incorrectly predicted as OV and BRCA. In Figure 4A, 14.00% of UCEC samples are predicted as OV, while 11.06% of UCECsamples are predicted to be BRCA. In Figure 4B, 14.45 and 11.74% of UCEC samples are predicted as OV and BRCA respectively. In Figure 4C, 13.32 and 12.42% of UCEC samples are predicted as OV and BRCA respectively. The reasons may be that UCEC, OV and BRCA are hormone-dependent tumors and they relate closely in tumorigenesis. The 16 and 27 risk regions were identified by an independent genome-wide association study (GWAS) on endometrial cancer and ovarian cancer, respectively (Glubb et al., 2020). Studies have shown that mutations in breast cancer susceptibility genes (BRCA1, BRCA2) have a relationship in hereditary ovarian cancer. Mutations at either end of the BRCA1 gene increase a person's risk of breast cancer, and its probability is higher than ovarian cancer. However, mutations in the middle of the BRCA1 gene put a person at a higher risk of ovarian cancer than breast cancer (Shi et al., 2017). In addition, there is also a study indicated that UCEC, OV and BRCA all have a relationship with the changes in estrogen and estrogen receptors (Rodriguez et al., 2019).

## Conclusions

In this paper, we introduce a gene selection algorithm—CRIA. We firstly apply this algorithm to 5 datasets and verify the effective performance of CRIA through comparison with other eight gene selection algorithms. The proposed algorithm can select features which are closely related to the class label. Then, we use this algorithm to select 200 genes that have a close relationship with cancer types from 24,174 genes features based on the value of copy number variations in the samples, and then combine three common classifiers—CatBoost, SVM and LightGBM to predict the type of cancer. Our experimental results show that our methods have higher accuracies than the state-of-the-art methods for solving this problem. Our research has a certain degree of interpretability for cancer-related researches at the genetic level. As we all know, cancer is closely related to gene structural variations and the appearance of cancer is often accompanied by abnormalities in the deoxyribonucleic acid (DNA) sequence. Because CNVs is one of the most crucial structural variations of genes, studying the relationship between cancers and CNVs is of great significance. Many studies have tried to utilize the genetic information of cancers to predict cancer type, which can provide significant guidance for patient care and cancer therapy in promptly.

The future direction of this work can continue to develop from two aspects. First of all, because we only use the datasets of six cancer types and the total number of samples is only 3,480 in this paper, by collecting data sets of other cancer types and optimizing the proposed algorithm, we can continue to conduct further research in the field of cancer classification based on copy number variations. Moreover, integrating non-CNVs features for the samples can be taken into consideration. In addition to using CNVs for cancer prediction, we can also apply other genetic information for cancer prediction, or combine several biomarkers to reduce the error rate of classification as much as possible.

## Data Availability Statement

The original contributions presented in the study are included in the article/supplementary material, further inquiries can be directed to the corresponding author/s.

## Author Contributions

QW conducted the experiments and wrote the manuscript. DL conceived and provided the main direction of the manuscript and guided the writing and modification of this manuscript. Both authors read and approved the manuscript.

## Funding

This work was supported by the National Natural Science Foundation of China (Grant No. 11571009) and Applied Basic Research Programs of Shanxi Province (Grant No. 201901D111086).

## Conflict of Interest

The authors declare that the research was conducted in the absence of any commercial or financial relationships that could be construed as a potential conflict of interest.

## Publisher's Note

All claims expressed in this article are solely those of the authors and do not necessarily represent those of their affiliated organizations, or those of the publisher, the editors and the reviewers. Any product that may be evaluated in this article, or claim that may be made by its manufacturer, is not guaranteed or endorsed by the publisher.
